# Emerging Roles of Metallothioneins in Beta Cell Pathophysiology: Beyond and above Metal Homeostasis and Antioxidant Response

**DOI:** 10.3390/biology10030176

**Published:** 2021-02-26

**Authors:** Mohammed Bensellam, D. Ross Laybutt, Jean-Christophe Jonas

**Affiliations:** 1 Pôle D’endocrinologie, Diabète et Nutrition, Institut de Recherche Expérimentale et Clinique, Université Catholique de Louvain, B-1200 Brussels, Belgium; jean-christophe.jonas@uclouvain.be; 2 Garvan Institute of Medical Research, Sydney, NSW 2010, Australia; r.laybutt@garvan.org.au; 3 St Vincent’s Clinical School, UNSW Sydney, Sydney, NSW 2010, Australia

**Keywords:** metallothionein, pancreatic beta-cell, insulin secretion, beta-cell compensation, beta-cell decompensation, stress response, diabetes

## Abstract

**Simple Summary:**

Defective insulin secretion by pancreatic beta cells is key for the development of type 2 diabetes but the precise mechanisms involved are poorly understood. Metallothioneins are metal binding proteins whose precise biological roles have not been fully characterized. Available evidence indicated that Metallothioneins are protective cellular effectors involved in heavy metal detoxification, metal ion homeostasis and antioxidant defense. This concept has however been challenged by emerging evidence in different medical research fields revealing novel negative roles of Metallothioneins, including in the context of diabetes. In this review, we gather and analyze the available knowledge regarding the complex roles of Metallothioneins in pancreatic beta cell biology and insulin secretion. We comprehensively analyze the evidence showing positive effects of Metallothioneins on beta cell function and survival as well as the emerging evidence revealing negative effects and discuss the possible underlying mechanisms. We expose in parallel findings from other medical research fields and underscore unsettled questions. Then, we propose some future research directions to improve knowledge in the field.

**Abstract:**

Metallothioneins (MTs) are low molecular weight, cysteine-rich, metal-binding proteins whose precise biological roles have not been fully characterized. Existing evidence implicated MTs in heavy metal detoxification, metal ion homeostasis and antioxidant defense. MTs were thus categorized as protective effectors that contribute to cellular homeostasis and survival. This view has, however, been challenged by emerging evidence in different medical fields revealing novel pathophysiological roles of MTs, including inflammatory bowel disease, neurodegenerative disorders, carcinogenesis and diabetes. In the present focused review, we discuss the evidence for the role of MTs in pancreatic beta-cell biology and insulin secretion. We highlight the pattern of specific isoforms of MT gene expression in rodents and human beta-cells. We then discuss the mechanisms involved in the regulation of MTs in islets under physiological and pathological conditions, particularly type 2 diabetes, and analyze the evidence revealing adaptive and negative roles of MTs in beta-cells and the potential mechanisms involved. Finally, we underscore the unsettled questions in the field and propose some future research directions.

## 1. Introduction

Type 2 diabetes (T2D) is a complex metabolic disorder involving the interaction of predisposing genetic factors and environmental risk elements, and pancreatic beta-cell failure is central to the development of the disease. Indeed, while peripheral insulin resistance is a key clinical predictor of T2D, progression to frank diabetes mellitus requires a further impairment of insulin secretion. In support of this view, non-diabetic obese-insulin resistant subjects can cope with the important metabolic demand by enhanced insulin synthesis and secretion and increased beta-cell mass, thereby preserving normal blood glucose levels at the cost of hyperinsulinemia. This stage is termed beta-cell adaptation or compensation. However, in a subpopulation of obese subjects, this phase is overtaken by a second phase where beta-cells fail to sustain an optimal secretory function, thereby leading to hyperglycemia. The ensuing vicious cycle of glucotoxicity contributes to further alterations of the beta-cell differentiated phenotype and the progressive worsening of the disease overtime. This second phase is termed beta-cell decompensation (these concepts are extensively reviewed in [1,2,3,4,5]). While the magnitude of the compensatory response is thought to be predetermined genetically, the precise molecular mechanisms involved in the progression from successful beta-cell compensation to beta-cell decompensation are poorly understood. A key challenge in diabetes research is the identification and characterization of these mechanisms towards a better understanding of beta-cell pathophysiology, and the development of novel targeted therapeutic strategies to preserve and/or restore the functional beta-cell mass in (pre)T2D subjects.

We have unveiled a novel role of metallothionein 1 (MT1) in beta-cell biology as a negative regulator of glucose-stimulated insulin secretion (GSIS) [6]. Using complementary models and in vivo and ex vivo/in vitro evidence, we demonstrated that *Mt1* inhibition potentiated GSIS and improved glucose tolerance, while *Mt1* overexpression attenuated the secretory response. These novel findings, together with previous findings in our research field and from other medical research fields, converge to underscore emerging pathophysiological roles of MTs that are beyond and above their classical beneficial involvement in metal homeostasis and antioxidant defense.

In this focused review, we introduce the MT gene family and their characterized biological roles. We then focus on MT gene expression, regulation, and roles in beta-cells. We describe the evidence showing positive effects of MTs on the beta-cell phenotype as well as the emerging evidence revealing negative effects. We also discuss the potential mechanisms underlying the negative effects of MTs on the beta-cell phenotype and expose in parallel findings from other medical research fields. Finally, we underscore unsettled questions and propose some future research directions needed to understand the complex role of MTs in beta-cell pathophysiology.

## 2. Metallothioneins: The Guardians of Metal and Redox Homeostasis

MTs are highly conserved proteins from prokaryotes to higher vertebrates. In mice, there are four MT isoforms encoded by *Mt1*, *Mt2*, *Mt3* and *Mt4* genes located on chromosome 8. In humans, there are eight different active *MT1* genes (*MT1A*, *MT1B*, *MT1E*, *MT1F*, *MT1G*, *MT1M*, *MT1X*) in addition to *MT2* (usually known as *MT2A*), *MT3* and *MT4* for a total of 11 functional MT isoforms (Table 1). The human MT genes are located on chromosome 16. While the expression of *Mt1* and *Mt2* is ubiquitous, *Mt3* is mainly expressed in neuronal cells and *Mt4* in squamous epithelium cells. Despite important sequence homology, several lines of evidence suggest specific regulatory mechanisms and roles of distinct MT isoforms within various contexts and cell types.

**Figure 1:**
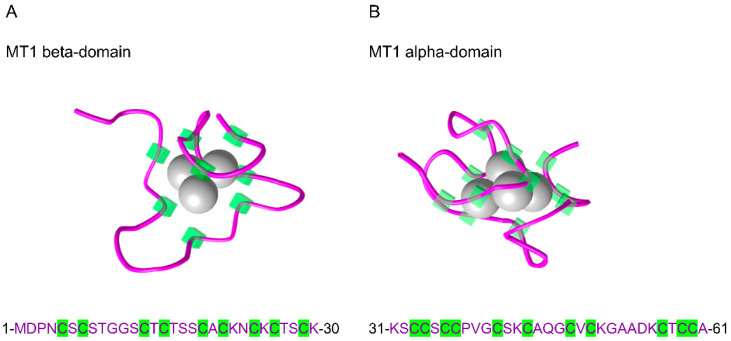
Three-dimensional solution structure of mouse [Cd7]-metallothionein 1 (MT1) obtained by NMR spectroscopy. (**A**) Mouse MT1 beta-domain with 3 Cd atoms and (**B**) mouse MT1 alpha-domain with 4 Cd atoms. The amino acid sequence is indicated below each domain structure. The protein sequence is represented in purple, and the cysteine residues are highlighted in green. Data were obtained from the NCBI’s Molecular Modeling Database (MMDB ID 12319 and 12320).

The MT proteins present unique molecular features. First, they have a low molecular mass of about 7 kDa. Second, they are rich in cysteine residues. For example, mouse MT1 is composed of 61 amino acids, of which 19 are cysteines (about 31%) (Figure 1A,B). Third, because of this important thiol content, MTs are able to bind different heavy metal elements, both essential, such as zinc (Zn) and copper (Cu), and toxic, such as cadmium (Cd). Evidence has demonstrated that vertebrate MTs exhibit a specific 3D structure with two distinct functional domains that bind divalent metals. For example, mouse MT1 has an alpha domain and a beta domain that can bind four and three divalent metal atoms, respectively (Figure 1A,B).

Owing to these characteristics, MTs play a fundamental role in heavy metal detoxification through the chelation of these elements and the reduction of their intracellular levels. Indeed, MT has been identified for the first time in the equine renal cortex as a Cd-rich protein [7,8], and mice lacking *Mt1* and *Mt2* genes displayed increased sensitivity to Cd and other heavy metal toxicity [9,10,11,12,13,14]. In contrast, mice overexpressing *Mt1* were protected against Cd lethality and hepatotoxicity [15]. MT gene promoters enclose metal response element (MRE) motifs [16,17], and their expression is markedly induced by heavy metals. Consequently, MTs have been proposed as useful biomarkers of metal exposure [18,19,20,21,22]. On the other hand, MTs also play key roles in physiological metal homeostasis, in particular Zn homeostasis. Zn is a trace element involved in key cellular processes, including insulin crystallization and storage within the secretory granules [23,24,25], and an important cofactor necessary for the proper function of about 10% of human proteins, including various enzymes and transcription factors [26]. Together with different Zn transporters, MTs are involved in the tight and dynamic regulation of intracellular Zn availability via the processes of chelation/storage, transfer, and release [27,28].

MTs are also involved in antioxidant defense and the regulation of cellular redox homeostasis. Through their sulfhydryl groups, they can scavenge reactive oxygen and nitrogen species (ROS, RNS) [29,30,31]. A subsequent release of Zn from the oxidized proteins leads to the activation of metal responsive transcription factor 1 (MTF1), binding to MREs, and ensuing upregulation of MT gene expression. Moreover, evidence suggested the involvement of oxidized glutathione in Zn release from MT [32], and a role of Zn in glutathione biosynthesis has also been proposed [33]. Zn is also a cofactor of the antioxidant enzymes superoxide dismutase 1 (SOD1) and SOD3, and evidence has shown the possible transfer of Zn from MT to SOD1 [34]. Besides MTF1, MT gene promoters also encompass antioxidant response element (ARE) motifs and can thus be regulated under oxidative stress conditions by other transcription factors [17,35,36,37]. Noteworthy, in comparison to reduced glutathione on a molar basis, the antioxidant activity of MT has been shown to be about 50 times higher against oxidative DNA damage and about 10 times higher against lipid peroxidation [38], and corroborative evidence supported the protective role of MTs against oxidative injury in various experimental models [30,39,40,41,42,43,44,45,46,47,48,49,50,51].

Moreover, MTs have been shown to play complex immunomodulatory roles and have been connected to inflammation and defense against infection [52]. While the precise pathways involved are only partially identified, it has been proposed that MT regulation of immune cell development and function may depend, at least in part, on their metal handling properties and antioxidant function [53].

In pancreatic beta-cells, oxidative stress is a central mechanism involved in the alteration of the beta-cell differentiated phenotype under chronic hyperglycemia [3,54,55,56,57]. Moreover, metal ions play essential physiological roles in beta-cells, and metal dyshomeostasis has been linked with the beta-cell demise in experimental models and was associated with diabetes and its complications in human subjects [58,59,60,61,62,63]. Interestingly, several clinical and epidemiological reports have shown that both type 1 diabetes (T1D) and T2D states are characterized by hypozincemia [64,65,66,67,68,69]. Furthermore, polymorphisms in *MT1A* and *MT2A* genes have been associated with increased risk for T2D and diabetic complications in humans [70,71,72]. However, while several studies have shown protective effects of MTs under different stress conditions both in vitro/ex vivo and in vivo, especially in the models of chemically induced diabetes and in the context of islet transplantation, emerging evidence, in contrast, revealed negative effects of MTs on beta-cell function and diabetes development via noncanonical mechanisms.

Before discussing and analyzing this emerging evidence, it is important to first describe the pattern of MT gene expression in rodents and human beta-cells.

## 3. Metallothioneins in Pancreatic Beta Cells

### 3.1. Expression of Metallothionein Genes in Mouse and Human Beta Cells

**Figure 2:**
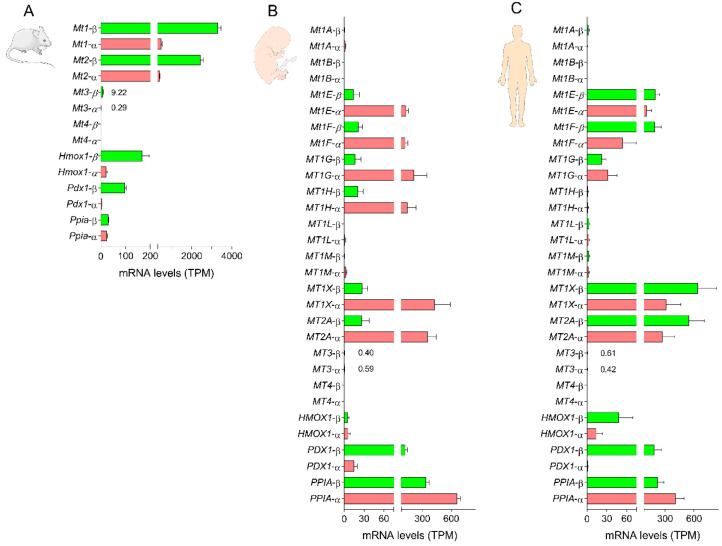
Pattern of metallothionein gene expression in pancreatic beta and alpha cells. (**A**) Changes in the mRNA levels of *Mt1-4*, *Hmox1*, *Pdx1* and *Ppia* in purified mouse beta and alpha cell preparations. Data are the means of the average number of transcripts per million (TPM) ± SEM for 5 (beta) and 3 (alpha) preparations. RNA sequencing data were obtained from the Gene Expression Omnibus database under the accession number GSE80673 [77]. (**B**) Changes in the mRNA levels of *MT1A*, *MT1B*, *MT1E*, *MT1F*, *MT1G*, *MT1M*, *MT1X*, *MT2A*, *MT3*, *MT4*, *HMOX1*, *PDX1* and *PPIA* in purified human fetal beta and alpha cell preparations. (**C**) Changes in the mRNA levels of *MT1A*, *MT1B*, *MT1E*, *MT1F*, *MT1G*, *MT1M*, *MT1X*, *MT2A*, *MT3*, *MT4*, *HMOX1*, *PDX1* and *PPIA* in purified human adult beta and alpha cell preparations. Data are the means of the average number of TPM ± SEM for 5 (fetal alpha), 6 (adult alpha and fetal beta) and 7 (adult beta) preparations. RNA sequencing data were obtained from the Gene Expression Omnibus database under the accession number GSE67543 [78].

The presence of constitutive and/or inducible MT expression in mouse and human islets has been known for decades [73,74,75,76]. Analysis of more recent RNA sequencing data of purified mouse islet cell preparations revealed that *Mt1* and *Mt2* were strongly expressed in beta-cells in comparison to the stress response gene *Hmox1*, the beta-cell enriched gene *Pdx1* or the house-keeping gene *Ppia,* for example, with *Mt1* exhibiting relatively higher mRNA levels than *Mt2*. On the other hand, *Mt3* was expressed to a much lower extent, while *Mt4* mRNAs were not detected (Figure 2A). We observed a similar expression pattern in whole mouse islets [6]. In alpha cells, *Mt1* and *Mt2* were also markedly expressed, yet without a difference between *Mt1* and *Mt2* and to a lower level in comparison to beta-cells, while *Mt3* was barely detectable and *Mt4* was absent (Figure 2A). The elevated basal expression of *Mt1* and *Mt2* in mouse beta-cells suggests the potential essential biological roles of these genes in insulin-secreting cells.

In humans, analysis of RNA sequencing data of purified islet cell preparations from fetal and adult donors revealed interesting findings. Thus, in immature fetal cells, *MT1A*, *MT1B*, *MT1L*, *MT1M*, *MT3* and *MT4* were either barely expressed or absent in both alpha and beta-cells. In contrast, *MT1E*, *MT1F*, *MT1G*, *MT1H*, *MT1X* and *MT2A* were expressed in both cell types, but about 6–16 times higher in fetal alpha vs. beta-cells (Figure 2B). Strikingly, in adult mature beta-cells, *MT1E*, *MT1F*, *MT1X* and *MT2A* mRNA levels were strongly upregulated by about 9–23-fold in comparison to fetal beta-cells and were also slightly higher in comparison to adult alpha cells (Figure 2C). Such maturity-dependent regulation appears to be unique to beta-cells since there was only a minor decrease in the expression of these genes in adult vs. fetal alpha cells. On the other hand, *MT1G* mRNA levels were slightly increased in adult beta-cells and markedly downregulated in adult alpha cells in comparison to fetal cells, while *MT1H* expression was strongly repressed in both cell types (Figure 2C). These observations underscore the specific pattern of MT gene expression in human alpha and beta-cells and suggest that *MT1E*, *MT1F*, *MT1X* and *MT2A* are important traits of differentiated mature human beta-cells and that they may play significant biological roles.

Ours and others’ evidence has demonstrated that MT gene expression in beta-cells is regulated by various physiological and stress stimuli, including metals and glucose.

### 3.2. Physiological and Pathological Regulation of MT Gene Expression in Beta Cells

#### 3.2.1. Regulation by Metals

As explained in Section 2, it is well established that MTs are highly responsive to physiological and toxic metal exposure in different organ systems and cell types. Unsurprisingly, this is also the case in pancreatic islets and beta-cells. Thus, earlier evidence has shown that Cd administration upregulated MT protein levels in the mouse pancreas [73]. In agreement, exposure of the mouse beta-cell line MIN6 to Cd augmented MT1 and MT2 protein levels [79]. Moreover, Zn exposure markedly increased *Mt1* mRNA levels in mouse, rat and chicken pancreatic endocrine and exocrine cells [80]. Furthermore, intraperitoneal injection of Zn salts in mice upregulated MT protein levels in pancreatic islets [74,75]. Similarly, exposure of mouse and rat islets to Zn salts ex vivo strongly increased *Mt1* and *Mt2* mRNA and protein levels [81,82,83]. In the rat beta-cell line INS1E, the mRNA levels of *Mt1a*, but not *Mt3*, were upregulated by ZnCl_2_ treatment and reduced by Zn chelation [84].

Thus, congruent evidence has demonstrated the regulation of *Mt1* and *Mt2* gene expression in pancreatic beta-cells in vitro/ex vivo and in vivo by metal exposure. As will be discussed in Section 4, this regulatory mechanism has been exploited to protect beta-cells from injury in some experimental models. Besides metals, MT gene expression in pancreatic beta-cells is also regulated by glucose.

#### 3.2.2. Regulation by Glucose

Glucose is the key driver of beta-cell function and homeostasis. Indeed, regular physiological stimulation by glucose and other nutrients is necessary to maintain the beta-cell differentiated phenotype and preserve beta-cell responsiveness to the next glucose challenge [85]. Conversely, prolonged exposure to under- or supra-physiological glucose levels negatively affects the beta-cell differentiated phenotype in parallel with the activation of various stress response pathways, impaired GSIS, and increased cell death [3,55,86].

**Figure 3:**
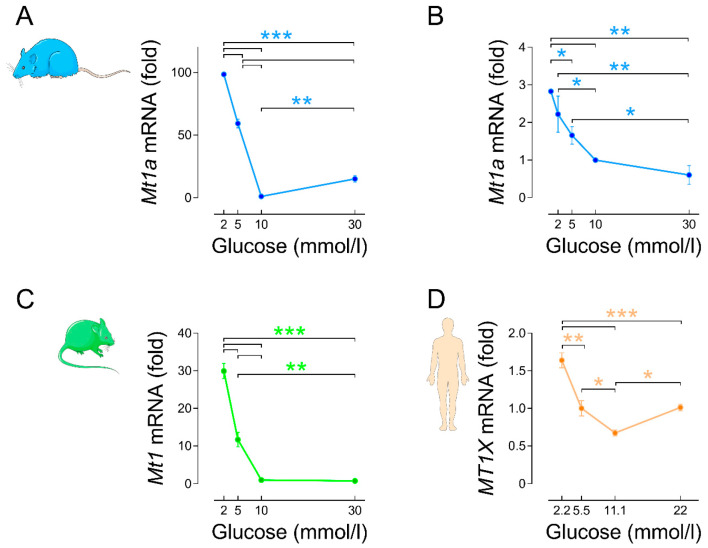
*Mt1a*, *Mt1* and *MT1X* mRNA levels are downregulated by glucose stimulation. (**A**) Changes in the mRNA levels of *Mt1a* in rat islets cultured for 18 h in the presence of increasing glucose concentrations (G2, G5, G10, G30). Islet isolation and culture, RNA extraction and RT-real time PCR were performed as previously described [87]. The primers used for *Mt1a* and the housekeeping gene *Tbp* are the same as in [87]. Data are means ± SEM of *n* = 4 experiments. (**B**) Changes in the mRNA levels of *Mt1a* in the rat beta-cell line INS 832/13 cultured for 24 h in the presence of increasing glucose concentrations (G0.5, G2, G5, G10, G30). INS 832/13 cells were kindly provided by Dr. Christopher Newgard, Duke University. Cell culture, RNA extraction and real-time PCR were performed as previously described [91]. Data are means ± SEM of *n* = 3 experiments. (**C**) Changes in the mRNA levels of *Mt1* in mouse islets cultured for 24 h in the presence of increasing glucose concentrations (G2, G5, G10, G30). Data were taken from [6] with permission of the publisher. Data are means ± SEM of *n* = 4 experiments. (**D**) Changes in the mRNA levels of *MT1X* in human islets from non-diabetic donors cultured for 24 h in the presence of increasing glucose concentrations (G2.2, G5.5, G11.1, G22). Data were taken from [6] with permission of the publisher. Data are means ± SEM of *n* = 3 experiments. * *p* < 0.05, ** *p* < 0.01, *** *p* < 0.001 for the indicated comparisons; one-way ANOVA with Newman–Keuls post hoc test.

We have previously found that *Mt1a* and *Mt2a* (the equivalent of mouse *Mt1* and *Mt2*) were among the 10 most-affected glucose-regulated genes in the transcriptome of rat islets [87]. Interestingly, *Mt1a* and *Mt2a* mRNA levels were strongly downregulated by 18 h culture in the presence of 10 mmol/L glucose (G10; the optimal concentration for rodent islet function and survival ex vivo [87,88,89,90]) instead of G2-5 (low non-stimulatory of insulin secretion). On the other hand, they were upregulated by culture in G30 vs. G10, thereby displaying an asymmetric V-shape expression profile with stronger downregulation (G10 vs. G2-5) than upregulation (G30 vs. G10) [87] (Figure 3A).

This upregulation is transient since no significant difference was observed after prolonged culture under G30 in comparison to G10 [83]. We also confirmed the glucose-dependent inhibition of *Mt1a* gene expression in the rat beta-cell line INS1 832/13 (Figure 3B). In mouse islets, *Mt1* and *Mt2* mRNA and protein levels were also markedly downregulated by culture in G10-30 vs. G2-5 [6,82] (Figure 3D). In contrast, *Mt3* mRNA levels were increased by glucose stimulation in rat and mouse islets [6,87]. In human islets, we found that glucose also downregulated *MT1E*, *MT1X* and *MT2A* mRNA levels in a concentration-dependent manner after culture in the presence of G2.2, G5.5 (the optimal concentration for human islets ex vivo [92,93]) and G11.1. Culture in the presence of G22 vs. G11.1 slightly upregulated *MT1E* and *MT1X* mRNA levels to a similar extent as with G5.5 [6] (Figure 3E). Collectively, these observations indicate that glucose stimulation overall inhibits MT gene expression in rodent and human islets. Noteworthy, this effect is in an inverse relationship with the stimulation of insulin secretion.

In contrast to negative glucose regulation, evidence has demonstrated that MT gene expression in beta-cells is markedly induced by various stress stimuli, including oxidative stress, proinflammatory cytokines, endoplasmic reticulum (ER) stress, and hypoxia.

#### 3.2.3. Regulation by Stress Stimuli

Earlier studies have shown that MT expression in mouse islets is upregulated ex vivo and in vivo by streptozotocin (STZ) treatment [74,81]. STZ is a glucosamine-nitrosourea alkylating agent that is particularly toxic to beta-cells [94], most likely via increased ROS generation and subsequent oxidative damage [95,96,97,98]. In agreement, we observed that exposure of rat islets to H_2_O_2_ markedly increased *Mt1a* mRNA levels [86]. Oxidative stress is a master mechanism involved in beta-cell demise under diabetes, not only because of the important macromolecular damage induced but also because ROS and RNS can activate other deleterious stress pathways. Alternatively, the activation of different stress pathways can lead to oxidative stress, and inflammation is one of these pathways.

Thus, we found that rat islet *Mt1a* mRNA levels were strongly upregulated by treatment with the proinflammatory cytokine IL1β [86]. MT protein levels were also upregulated by IL1β treatment in rat islets and beta-cell lines and by the combination of IL1β + TNFα in mouse islets [99]. Similarly, analysis of the transcriptome of purified rat beta-cells exposed to the proinflammatory cytokines IL1β, IL1β + IFNγ or TNFα + IFNγ also revealed marked and time-dependent upregulation of *Mt1a* mRNA levels [100,101]. Upregulation of *Mt1a* by proinflammatory cytokines may involve oxidative stress [102,103]. Interestingly, in the latter transcriptomics studies, upregulation of *Mt1a* mRNA levels was paralleled by upregulation of other oxidative stress-responsive genes, including catalase, several glutathione s-transferase genes and *Sod2* [100,101]. In contrast, MT induction by IL1β in the mouse beta-cell line βHC9 was not altered by inducible nitric oxide synthase inhibitors, while the NO generator sodium nitroprusside failed to significantly affect MT protein levels thereby suggesting that NO may not play a significant role in MT induction [99]. On the other hand, MT gene promoters enclose signal transducer and activator of transcription (STAT) binding sites and may thus be induced via this pathway upon cytokine stimulation in beta-cells [17,104].

Besides cytokines, evidence indicated that MT gene expression is also induced by glucocorticoid hormones. Thus, mouse *Mt1* and *Mt2* and several human MT gene promoters have been shown to enclose glucocorticoid response element (GRE) motifs [17,105,106]. In rodent islets and insulinoma cell lines, dexamethasone treatment time-dependently upregulated MT protein levels and displayed a synergistic effect when combined with cytokines [99].

Evidence also has suggested a role for lipotoxicity in the regulation of MT gene expression. Thus, exposure of human islets to 500 µmol/L palmitate acutely and transiently upregulated the mRNA levels of several MT genes [107]. Moreover, chronic exposure of human islets to 400 µmol/L oleates increased *MT1F* mRNA levels in association with increased ROS generation and upregulation of catalase [108]. Elevated levels of free fatty acids (FFAs) may induce beta-cell oxidative stress, likely via a mechanism involving peroxisomal H_2_O_2_ generation [109]. Alternatively, it is well established that FFAs trigger beta-cell ER stress [110]. In support of the role of ER stress in the regulation of MT gene expression, we found that the pharmacological ER stress inducer thapsigargin strongly increased the mRNA levels of *Mt1a* in rat islets [86].

Rodent and human islet MT gene expression is also regulated by hypoxic stress. Thus, rat islet *Mt1a* mRNA levels were significantly upregulated by culture in the presence of 5% instead of 20% O_2_ [86]. A stronger effect was observed for *Mt1* when mouse islets were cultured in the presence of 1% vs. 20% O_2_ [6]. Likewise, mouse islet *Mt2* mRNA levels were upregulated by hypoxia, while, in contrast, *Mt3* mRNA levels were downregulated [111]. In human islets, hypoxia (1% O_2_) also markedly upregulated *MT1A* and, to a stronger extent, *MT2A* mRNA levels [111].

These types of stress characterize the (pre)diabetic milieu, and some of them are already present at the stage of beta-cell compensation in *db*/*db* mice, including oxidative stress and inflammation [112]. However, unexpectedly, we found that *Mt1* and *Mt2* are differentially regulated in conditions of beta-cell compensation and failure in vivo.

#### 3.2.4. Regulation during Beta Cell Compensation and Failure

To examine the temporal changes of islet *Mt1* and *Mt2* gene expression under the states of beta-cell compensation and failure, we used two mouse models of obesity with an opposite propensity for the development of diabetes: (1) the diabetes resistant *ob*/*ob* mice on the C57BL/6J genetic background and (2) the diabetes-prone *db*/*db* mice on the C57BL/KsJ genetic background, which exhibit a progressive age-dependent beta-cell decompensation and development of hyperglycemia [112,113,114]. Thus, we found that *Mt1* and *Mt2* mRNA levels were markedly decreased in the islets of compensating *ob*/*ob* mice at 6 and 16 weeks of age vs. age-matched lean control mice. Interestingly, in *db*/*db* mice, islet *Mt1* and *Mt2* mRNA levels were similarly decreased in 6-week-old prediabetic mice (compensation) but were increased in 16-week-old diabetic mice (beta-cell failure) in comparison to age-matched lean control mice [6]. Moreover, we found that increased plasma insulin levels (beta-cell compensation) correlated with a strong downregulation of *Mt1* and *Mt2* mRNA levels in the islets of diet-induced obese (DIO) mice (6 weeks of high-fat diet) [6].

In humans, a previous study examining the transcriptome of islet tissue obtained by laser capture microdissection from non-diabetic subjects and subjects with T2D revealed a significant upregulation of *MT1E*, *MT1M*, *MT1X* and *MT2A* mRNA levels in the diabetic group. In this study, *MT1E*, *MT1X* and *MT2A* displayed the strongest array signals among the MT gene isoforms [115]. In agreement, we subsequently found a significant increase in *MT1X* mRNA levels in the islets of donors with T2D vs. non-diabetic donors [6].

While upregulation of islet MT genes under diabetes may be explained by an effect of the unfavorable glucolipotoxic and proinflammatory diabetic milieu, the downregulation of islet *Mt1* and *Mt2* mRNA levels in compensating 6-week-old *db*/*db* mice and DIO mice, despite the presence of an evident stress signature in these islets [112,116,117], is intriguing and suggests a different regulatory mechanism and a biological role other than antioxidant defense in this context.

Collectively, these in vivo findings highlight a negative correlation between beta-cell compensation and *Mt1*-*Mt2* gene expression. Taken together with the inhibitory effect of glucose on islet MT gene expression ex vivo, these observations raise the hypothesis that changes in *Mt1* and/or *Mt2* gene expression may play a role in the modulation of beta-cell function. However, if upregulation of MT gene expression has a negative impact on beta-cell function, then upregulation of MT gene expression in diabetes may play both adaptive and negative roles.

## 4. Adaptive Roles of MTs in Beta Cells

Past evidence has shown that exposure of isolated mouse islets to Cd reduced GSIS. However, when mice were pretreated with Cd prior to islet isolation which upregulated MT protein levels in the pancreas, subsequent exposure of isolated islet to Cd did not reduce GSIS. This was the first study suggesting a protective role of MT in beta-cell against acute Cd toxicity [73]. Similarly, pretreatment of mice with Zn-enriched drinking water increased islet MT protein levels and partially protected against diabetes induced by multiple low doses of STZ [75]. In isolated rat islets, supplementation of culture medium with ZnCl_2_ markedly augmented *Mt1a* and *Mt2a* gene expression and significantly reduced beta-cell apoptosis induced by prolonged culture in the presence of G5 or G30 instead of G10 [83]. Several Zn supplementation strategies have been tested in experimental models of diabetes as well as in humans [118]. Some of these studies revealed beneficial effects and improvement of glycemic control, while other studies showed no significant effect or even a negative effect [119,120,121,122]. Importantly, the observed beneficial effects in some of these studies cannot be ascribed with certainty to the induction of MTs given the pleiotropic effects of Zn, and hence transgenic mouse models have provided more solid insights about the protective actions of MTs in beta-cell.

Thus, in comparison to control FVB mice, transgenic mice overexpressing the human *MT2A* gene under the control of the human insulin promoter (*MT2A*-Tg) exhibited reduced hyperglycemia after STZ injection. Moreover, in comparison to control islets, transgenic islets showed reduced DNA fragmentation, NAD^+^ depletion, degranulation, and necrosis upon STZ treatment ex vivo [123]. When transferred on the C57BL/6 J background, these transgenic mice also displayed a marked reduction of hyperglycemia after STZ treatment [124]. Moreover, in response to acute exposure to various prooxidant agents, *MT2A*-Tg dispersed islet cells exhibited reduced intracellular ROS levels vs. control FVB islet cells. A similar result was obtained upon exposure of whole islets to hypoxia (1% O_2_) in association with higher cell viability in *MT2A*-Tg islets. In agreement, when transplanted into diabetic mice, *MT2A*-Tg islets extended the duration of euglycemia, and transgenic islet grafts exhibited reduced nitrotyrosine staining and higher insulin staining. Furthermore, NO-induced damage was also attenuated in *MT2A*-Tg islets vs. control FVB islets ex vivo [125]. In contrast, *Mt1*-*Mt2*-KO islets showed a higher cell death rate under hypoxia (1% O_2_) in comparison to control islets [111].

In parallel with transgenic mouse evidence, another strategy uses recombinant MT proteins fused with protein transduction domains (also known as cell-penetrating peptides). Owing to their positive charge, these peptides can bind negatively charged plasma membrane, thereby enhancing the intracellular delivery of the fusion protein. Thus, using a basic domain of HIV-1 Tat protein, Tat-MT1A fusion protein was developed and has been shown to slightly protect INS1 cells against glucolipotoxicity and hypoxia [126]. The same group showed in a subsequent study a protective effect of Tat-MT1A pretreatment against glucolipotoxicity in INS1 cells and rat islets. This effect was associated with reduced ROS production and nuclear factor κB activation [127]. In vivo, intraperitoneal injection of Tat-MT1A in mice showed possible delivery into islets, and mice receiving 8 injections of Tat-MT1A over 11 days were partially protected against diabetes induced by multiple low doses of STZ. Moreover, Tat-MT1A treatment every 3 days for 18 weeks slightly improved glucose tolerance in OLETF rats and delayed diabetes onset [127]. The combination Tat-MT1A + Tat-SOD1 also showed a protective effect against islet injury and delayed diabetes development in vivo [128]. In the context of islet transplantation, this strategy has been shown to enhance islet graft survival and glycemic control [128,129,130].

All in all, these cumulative findings reveal an important protective antioxidant role of MT in beta-cells and suggest that MT gene induction in the islets of diabetic animal models and human subjects may play a key adaptive role. Nevertheless, this evidence has been challenged by previous and recent emerging findings revealing negative roles of MTs in beta-cells.

## 5. Negative Roles of MTs in Beta Cells

Proinflammatory cytokines are involved in beta-cell demise in T1D and T2D, and this effect is mediated, at least in part, via increased ROS and RNS production and subsequent oxidative damage. In addition, infiltrating immune cells can also produce ROS and RNS, thereby exacerbating islet oxidative stress. Therefore, overexpression of MT has been tested as a strategy to protect beta-cells in the T1D model of non-obese diabetic (NOD) mice. These *MT2A*-Tg NOD mice displayed normal glucose tolerance before diabetes induction, and their islets had normal expression of ER stress genes (*Hspa5*, *Ddit3*) [131]. Surprisingly, in comparison to control NOD mice, *MT2A*-Tg NOD mice exhibited a marked acceleration of diabetes onset after cyclophosphamide injection (used to accelerate and synchronize diabetes development in this model). This was associated with reduced pancreatic insulin content and increased cleaved-caspase 3 immunostaining. Moreover, spontaneous diabetes onset was also accelerated in male, but not female, *MT2A*-Tg NOD mice. Furthermore, isolated *MT2A*-Tg NOD islets were more sensitive to the proinflammatory cytokines IL1β + IFNγ + TNFα. Thus, despite a clear attenuation of cytokine-induced ROS generation, these islets displayed increased cleaved-caspase 3 protein levels, reduced AKT and FOXO1 phosphorylation, reduced PDX1 protein levels, and reduced metabolic activity. The addition of vanadate to the culture medium preserved *MT2A*-Tg NOD islets shape and metabolic activity under cytokine treatment, thereby suggesting a potential role of protein tyrosine phosphatase activity in the sensitivity of *MT2A*-Tg NOD islets to proinflammatory cytokines [131]. Accelerated diabetes onset in NOD mice also has been triggered, though to a lesser extent, by catalase, but not *SOD2*, transgenic overexpression (no difference in the latter model) [131]. Nevertheless, in the same model, other studies have shown a protective effect of beta-cell transgenic overexpression of *Hmox1* [132] and islet overexpression of *Txn* [133] and *Sod2* [134]. This indicates that the negative impact of *MT2A* overexpression is unlikely to be related to ROS scavenging properties.

On the C57BL/6 J background, *MT2A*-Tg mice exhibited an age-dependent worsening of glucose tolerance in association with reduced plasma insulin levels during intraperitoneal glucose tolerance test (*ip*GTT), reduced pancreatic insulin content and reduced islet size. This phenotype was more pronounced in males than in females. Moreover, GSIS was abolished in the transgenic islets in comparison to control islets. Abrogation of GSIS in this model seems to result from an important alteration of the beta-cell differentiated phenotype. Indeed, the *MT2A*-Tg male islets displayed a marked reduction in the mRNA levels of several beta-cell enriched genes (*Ins1*, *Pdx1*, *Neruod1*, *Slc2a2*, *Gck*) in parallel with significant upregulation of stress response genes (*Hmox1*, *Trib3*, *Tnfrsf1b*) [124]. Beta-cell demise in this model may be a consequence of ER and/or oxidative stress. The latter may stem from the high expression level driven by the human insulin promoter and subsequent high biosynthesis rate of MT2A that exceeds the ER folding capacity. A role of (islet resident) macrophages or other immune cells is also possible given that MTs have chemokine-like actions and can activate immune cells, which may, in turn, affect beta-cells by producing proinflammatory cytokines and ROS/RNS (discussed in Section 6).

Although expressed to a much lesser degree in comparison to *Mt1* and *Mt2*, *Mt3* may also play a negative role in beta-cells. One study has shown that *Mt3*-KO mice were protected against STZ-induced diabetes and *Mt3*-KO islets were resistant to STZ and NO toxicity ex vivo. This effect has been proposed to involve a partial reduction of phosphodiesterase 3A expression [135]. However, other mechanisms may be involved. Moreover, it is unclear if these islets presented a compensatory upregulation of other MT gene isoforms.

On the other hand, given the differential expression of *Mt1* and *Mt2* in the contexts of beta-cell compensation and failure in vivo, and the inverse correlation between *Mt1*-*Mt2* gene expression and insulin secretion in isolated islets, we verified whether changes in *Mt1* and/or *Mt2* gene expression might have an impact on beta-cell function using a comprehensive approach and complementary models [6].

Thus, in *Mt1*-*Mt2*-KO mice on the Sv129 genetic background [10], we observed a significantly improved glucose tolerance during *ip*GTT in association with increased plasma insulin levels during the test. In agreement, an overnight fasting-1 h refeeding test revealed that *Mt1*-*Mt2*-KO mice displayed lower blood glucose levels after refeeding. Of note, *Mt1*-*Mt2*-KO mice showed a higher daily food intake in comparison to control mice. In parallel, insulin tolerance tests did not reveal a difference between control and *Mt1*-*Mt2*-KO mice, thereby suggesting that improved glucose tolerance in these mice stems from an effect of *Mt1*-*Mt2* deletion on insulin secretion and not insulin action. In support of this mechanism, isolated *Mt1*-*Mt2*-KO islets showed enhanced GSIS in comparison to control islets without a difference in islet insulin content. Furthermore, in MIN6 cells, *Mt1* knockdown, but not *Mt2*, also augmented GSIS, thereby underscoring the role of MT1 in the negative regulation of insulin secretion. Changes in cytosolic free calcium (Ca^2+^) and Zn^2+^ concentrations in response to acute stepwise increases in glucose levels were similar between control and *Mt1*-*Mt2*-KO islets. Similarly, changes in intracellular NAD(P)H levels in response to acute glucose stimulation were not different between the two types of islets, thereby suggesting that the enhanced secretory phenotype involves a mechanism downstream of glucose metabolism and Ca^2+^ influx. Importantly, besides glucose, the potentiation of insulin secretion in *Mt1*-*Mt2*-KO vs. control islets was also observed in response to 30 mmol/L potassium salt (KCl). High K^+^ levels depolarize the beta-cell plasma membrane and trigger insulin secretion independently from glucose metabolism [6]. Together, these findings point to a potential effect of *Mt1*-*Mt2* deletion on the secretory machinery.

Moreover, to assess whether *Mt1* overexpression results in the opposite phenotype to *Mt1* deletion, we used transgenic mice overexpressing mouse *Mt1* under the control of its natural promoter in a position-independent and copy-dependent manner on the C57BL/6 J background (*Mt1*-Tg) [136,137]. In comparison to *MT2A*-Tg mice [124], this model presents the advantage of overexpressing the mouse *Mt1* gene rather than the human *MT2A* gene. It is important here to emphasize that MT1 and MT2 are not always redundant and present differences at the level of amino acid sequence, protein–protein interactions as well as metal affinities, and hence exhibit functional differences as we saw in our study [138,139]. The use of the strong human insulin promoter and subsequently enhanced MT2A protein synthesis may explain, at least in part, the stress signature observed in *MT2A*-Tg islets [124]. Indeed, our *Mt1*-Tg islets displayed a normal expression of beta-cell enriched genes and stress response genes [6]. Moreover, *Mt1*-Tg mice had normal fed and fasted blood glucose levels and normal glucose tolerance. However, they displayed lower blood glucose levels during the insulin tolerance test, an effect that may result from an impact of MT1 overexpression on peripheral tissues. Importantly, isolated *Mt1*-Tg islet exhibited a significant attenuation of GSIS vs. control islets. This effect was independent of changes in islet insulin content and intracellular free Ca^2+^, Zn^2+^ and NAD(P)H levels, thereby further supporting the assumption that MT1 impacts GSIS at a level downstream of glucose metabolism and Ca^2+^ influx, in harmony with the results from *Mt1*-*Mt2*-KO islets [6].

**Figure 4:**
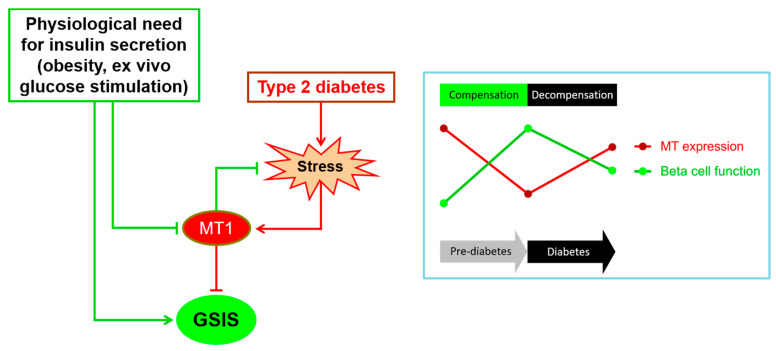
The proposed model of the role of MT1 in the pathogenesis of type 2 diabetes. Under physiological conditions requiring the stimulation of insulin secretion, *Mt1* gene expression is repressed, thereby allowing enhanced secretory response to meet the metabolic needs. In contrast, under diabetes conditions, the unfavorable diabetic environment and subsequent induction of oxidative stress and other stress pathways lead to the upregulation of *Mt1* gene expression. The latter is a double-edged sword that will, on one hand, counter beta-cell stress, at least in part through scavenging of ROS, and on the other hand, inhibit GSIS. These events occur sequentially during the natural history of diabetes, as depicted in the insert. In the prediabetic phase (beta-cell compensation), the beta-cell function is markedly enhanced in parallel with significant downregulation of MT gene expression. Conversely, in the diabetic phase (beta-cell decompensation), beta-cell function declines in parallel with significant upregulation of MT gene expression. GSIS: glucose-stimulated insulin secretion.

Taken together, these corroborative findings strongly support the involvement of MT1 in the negative regulation of insulin secretion in mouse beta-cells. Therefore, *Mt1* downregulation in obesity may be an important component of the beta-cell compensatory response. In contrast, its upregulation in T2D may contribute to impaired secretory function, although it may have a parallel antioxidant role (Figure 4). However, several questions remain unsettled.

## 6. Unsettled Questions and Future Research Directions

The precise molecular pathway(s) underlying the inhibitory action of MT1 on insulin secretion is(are) elusive at this stage. Our functional data support an impact on exocytosis. One plausible mechanism may involve an interaction of MT1 with component(s) of the secretory machinery to modulate insulin granule dynamics. In this regard, MT3 has previously been shown to interact with RAB3A and has been proposed to play a role in presynaptic vesicle trafficking [140]. MT3 has also been shown to interact with SEC84 (also known as Exo84p), a subunit of the exocyst that is involved in secretory vesicle targeting and docking at the plasma membrane [141,142]. An interaction between MT and α-synuclein has also been proposed [143]. The latter has been shown to play a role in the regulation of insulin secretion in beta-cells [144,145,146]. Most of the investigation in this area has focused on MT3-protein interactions in the central nervous system [138]. Therefore, further investigation is needed to decipher the precise interacting partners of MT1 in beta-cells. It would also be important to determine the precise subcellular localization of MT1 under non-stimulatory and glucose stimulatory conditions. These experiments will be important to better understand the role of MT1 in beta-cell biology. Unfortunately, because of important sequence homology between MT1 and MT2 proteins, reliable specific antibodies against MT1 are at present unavailable. The development of a specific MT1 antibody would be important not only for immunolocalization studies but also from a future therapeutic perspective.

In humans, it is currently unknown whether *MT1X* and/or other MT gene isoforms are affected in the islets of obese compensating subjects and how. However, noteworthy, evidence has shown downregulation of *MT1X* and *MT1G* expression in lymphoblastoid cells of male subjects with non-syndromic obesity in comparison to lean subjects [147]. Moreover, it is unclear whether MT1X (or another human MT isoform) plays a role in human beta-cells similar to that of MT1 in mouse beta-cells. Thus, further gene inhibition/overexpression studies in human beta-cell lines and primary islets are required to shed light on the potential involvement of MT1X in the inhibition of insulin secretion in humans.

Upregulation of islet MT gene expression in T2D is associated with alteration of the beta-cell dedifferentiated phenotype. The phenomenon of dedifferentiation has been proposed as an adaptive mechanism to mitigate cell death at the expense of insulin secretory dysfunction under specific diabetic stress conditions [55]. It is possible that MT contributes to this phenomenon via parallel attenuation of insulin secretion and protection against oxidative stress, but it is unclear whether beta-cell dedifferentiation plays a role upstream of MT gene induction.

Another big question concerns the mechanisms underlying the downregulation of *Mt1* and *Mt2* during beta-cell compensation. The potential involvement of metal dyshomeostasis in this regulation is not supported by a previous study that showed no elemental difference in the beta cells of prediabetic Chinese hamsters [148]. Additionally, it has been proposed that diabetes development in *db*/*db* mice was not associated with reliable elemental changes in beta-cells [149]. However, the use of the novel synchrotron X-ray fluorescence imaging system to compare the elemental profiles of whole cryofrozen islets from compensating and decompensating mice may reveal novel insights [150]. Otherwise, in other cell systems, several mechanisms have been proposed to repress MT gene expression, including transcriptional repressors and promoter methylation [17,151]. These mechanisms may play a role in the regulation of *Mt1* and *Mt2* under the states of beta-cell compensation and decompensation. Understanding these molecular events and potential upstream regulators (hormones, metabolism/metabolites, metals?) is important from a fundamental and translational point of view.

Similarly, the mechanism(s) involved in the downregulation of islet *Mt1* and *Mt2* gene expression by glucose stimulation ex vivo is (are) also unclear. Interestingly, pharmacological glucokinase activation under low glucose conditions also downregulated *Mt1a* mRNA levels in rat islets [152]. This indicates that it is not glucose per se that inhibits *Mt1a* gene expression but rather signals engendered by the acceleration of mitochondrial metabolism and/or the stimulation of insulin secretion. Reduced mitochondrial superoxide anion production upon acute glucose stimulation may also play a role [153,154,155]. Whether changes in metals are also involved is uncertain. However, we observed that the Zn transporter gene *Slc30a1* (also known as *Znt1*), which is involved in Zn efflux from the cell, is also markedly downregulated by glucose stimulation in parallel to *Mt1a* and *Mt2a*, which may reflect changes in Zn intracellular levels [87]. In addition, *Mt1a* and *Mt2a* mRNA upregulation by culture in G5 vs. G10 was abrogated by Zn chelation [83]. Prolonged culture in the presence of low glucose concentrations may trigger lysosomal insulin granule degradation [156]. Therefore, it is possible that insulin granule degradation under low nutrient conditions releases Zn ions that lead to upregulation of MT gene expression. In contrast, nutrient stimulation and subsequent increase in proinsulin biosynthesis may channel cellular Zn to the secretory granules for insulin crystallization and packaging, which may contribute to reduced MT gene expression. This tempting hypothesis is, however, in disagreement with the reported increase in cytosolic free Zn in mouse beta-cells cultured for 24h in G16.7 vs. G3 [82]. But since cellular Zn dynamics are complex and differences may exist between the different subcellular compartments, further investigation using complementary models and novel technological tools is needed to uncover the precise mechanisms involved in the downregulation of MT gene expression by glucose stimulation and determine if Zn plays a role or not. Besides Zn, several reports revealed Cu dyshomeostasis in T1D and T2D subjects [63,64,67,68,157,158]. Cu is an important cofactor for different enzymes, including SOD1 and mitochondrial cytochrome c oxidase. However, the presence of high concentrations can play a role in oxidative stress (Fenton reaction). Cu also induces MT gene expression, and MTs are involved in the regulation of Cu homeostasis [139]. In cultured rat islets, we previously observed that changes in the mRNA levels of the Cu high-affinity transporter *Slc31a1* (also known as *Ctr1*) in response to increasing glucose concentrations paralleled those of *Mt1a* and *Mt2a*, which may reflect parallel changes in Cu intracellular levels [87]. Therefore, the role of Cu in the regulation of MT gene expression under these conditions cannot be excluded.

Interestingly, MTs may also have a chemokine-like role. They have been shown to be present in different extracellular body fluids and potentially secreted by cells, although the mechanism involved is unknown (they do not have an N-terminal signal peptide sequence). They may also bind a receptor(s) on the surface of neuronal, renal and immune cells [159,160,161,162,163]. In the context of inflammatory bowel disease, MT1 and/or MT2 have been proposed to play the role of danger signals released by dead intestinal epithelial cells that attract and activate leukocytes. Indeed, *Mt1*-*Mt2*-KO mice displayed reduced severity of colitis in association with reduced leukocyte infiltration. Moreover, blockade of extracellular MT1 and MT2 with a monoclonal antibody reduced macrophage infiltration in control mice. In agreement, the Boyden chamber migration assay revealed that the attraction of leukocytes to dead intestinal epithelial cell supernatant was also abolished with the anti-MT1/MT2 antibody [164]. In our context, a previous clinical trial revealed that Zn supplementation in T2D patients reduced glucose disposal, increased fasting glucose and fructosamine levels, and increased the reactivity of their T-lymphocytes to phytohemagglutinin [119]. However, it is unclear if MT1 and/or MT2 play such chemokine-like roles in the islets of diabetic mice and human subjects. This is an attractive hypothesis that is compatible with the observed increase in islet macrophages in diabetic *db/db* mice, but not *ob/ob* compensating mice. Interestingly, macrophages from diabetic *db/db* islets also displayed a distinct proinflammatory gene expression pattern in comparison to those from *ob/ob* islets [165].

## 7. Conclusions

Contrary to the general perception that MTs are protective cellular effectors under stress conditions, several lines of evidence from in vitro/ex vivo models and animal studies have revealed complex negative roles of MTs in pancreatic beta-cells that are far from being completely understood. In light of this emerging evidence, the safety and usefulness of therapeutic strategies that augment MT levels in beta-cells should be considered with caution. More studies in human beta-cells and the development of novel animal models and technical tools are needed to fill the gaps of knowledge in this field and answer the unsettled questions. A better understanding of the role of MTs in beta-cell pathophysiology may lead to a novel targeted therapeutic strategy to preserve/enhance insulin secretion in obese (pre)diabetic subjects.

## Figures and Tables

**Table 1:** Human metallothionein (MT) protein sequences.

Protein	ID	AA	Protein Sequences
MT1A	P04731	61	MDPNCSCAT-GGSCTCTGSCKCKECKCTSCKKSCCSCCPMSCAKCAQGCICKGAS------EKCSCCA
MT1B	P07438	61	MDPNCSCTT-GGSCACAGSCKCKECKCTSCKKCCCSCCPVGCAKCAQGCVCKGSS------EKCRCCA
MT1E	P04732	61	MDPNCSCA-TGGSCTCAGSCKCKECKCTSCKKSCCSCCPVGCAKCAQGCVCKGAS------EKCSCCA
MT1F	P04733	61	MDPNCSCA-AGVSCTCAGSCKCKECKCTSCKKSCCSCCPVGCSKCAQGCVCKGAS------EKCSCCD
MT1G	P13640	62	MDPNCSCAAAGVSCTCASSCKCKECKCTSCKKSCCSCCPVGCAKCAQGCICKGAS------EKCSCCA
MT1H	P80294	61	MDPNCSCEA-GGSCACAGSCKCKKCKCTSCKKSCCSCCPLGCAKCAQGCICKGAS------EKCSCCA
MT1L	Q93083	61	MDPNCSCAT-GGSCSCASSCKCKECKCTSCKKSCCSCCPMGCAKCAQGCVCKGAS------EKCSCCA
MT1M	Q8N339	61	MDPNCSCTT-GVSCACTGSCTCKECKCTSCKKSCCSCCPVGCAKCAHGCVCKGTL------ENCSCCA
MT1X	P80297	61	MDPNCSCSPV-GSCACAGSCKCKECKCTSCKKSCCSCCPVGCAKCAQGCICKGTS------DKCSCCA
MT2A	P02795	61	MDPNCSCA-AGDSCTCAGSCKCKECKCTSCKKSCCSCCPVGCAKCAQGCICKGAS------DKCSCCA
MT3	P25713	68	MDPETCPCPSGGSCTCADSCKCEGCKCTSCKKSCCSCCPAECEKCAKDCVCKGGEAAEAEAEKCSCCQ
MT4	P47944	62	MDPRECVCMSGGICMCGDNCKCTTCNCKTYWKSCCPCCPPGCAKCARGCICKGGS------DKCSCCP

AA: amino acids. Conserved residues between all human MT proteins are highlighted in green. Sequence alignment was performed using the Align tool of the Uniprot database.

## Data Availability

Not applicable.
